# Hematopoietic stem cells and retroviral infection

**DOI:** 10.1186/1742-4690-7-8

**Published:** 2010-02-04

**Authors:** Prabal Banerjee, Lindsey Crawford, Elizabeth Samuelson, Gerold Feuer

**Affiliations:** 1 Department of Microbiology and Immunology, SUNY Upstate Medical University, Syracuse, NY, 13210, USA; 2 Center for Humanized SCID Mice and Stem Cell Processing Laboratory, SUNY Upstate Medical University, Syracuse, NY, 13210, USA

## Abstract

Retroviral induced malignancies serve as ideal models to help us better understand the molecular mechanisms associated with the initiation and progression of leukemogenesis. Numerous retroviruses including AEV, FLV, M-MuLV and HTLV-1 have the ability to infect hematopoietic stem and progenitor cells, resulting in the deregulation of normal hematopoiesis and the development of leukemia/lymphoma. Research over the last few decades has elucidated similarities between retroviral-induced leukemogenesis, initiated by deregulation of innate hematopoietic stem cell traits, and the cancer stem cell hypothesis. Ongoing research in some of these models may provide a better understanding of the processes of normal hematopoiesis and cancer stem cells. Research on retroviral induced leukemias and lymphomas may identify the molecular events which trigger the initial cellular transformation and subsequent maintenance of hematologic malignancies, including the generation of cancer stem cells. This review focuses on the role of retroviral infection in hematopoietic stem cells and the initiation, maintenance and progression of hematological malignancies.

## Introduction

Hematopoiesis is a highly regulated and hierarchical process wherein hematopoietic stem cells (HSCs) differentiate into mature hematopoietic cells [1]. It is a process controlled by complex interactions between numerous genetic processes in blood cells and their environment. The fundamental processes of self-renewal and quiescence, proliferation and differentiation, and apoptosis are governed by these interactions within both hematopoietic stem cells and mature blood cell lineages. Under normal physiologic conditions, hematopoietic homeostasis is maintained by a delicate balance between processes such as self-renewal, proliferation and differentiation versus apoptosis or cell-cycle arrest in hematopoietic progenitor/hematopoietic stem cells (HP/HSCs). Under stress conditions, such as bleeding or infection, fewer HP/HSCs undergo apoptosis while increased levels of cytokines and growth factors enhance proliferation and differentiation. In a normally functioning hematopoietic system, the kinetics of hematopoiesis return to baseline levels when the stress conditions end. Deregulation of the signaling pathways that control the various hematopoietic processes leads to abnormal hematopoiesis and is associated with the development of cancer, including leukemia (reviewed in [2]).

Although not fully characterized, deregulation of normal hematopoietic signaling pathways in HP/HSCs following viral infection has previously been documented [3-5]. Previous studies demonstrated productive infection of HP/HSCs by retroviruses and suggested that retroviral mediated leukemogenesis shares similarities with the development of other types of cancer, including the putative existence of cancer stem cells (CSCs) [6,7]. Here we discuss the evidence demonstrating that retroviruses can infect HP/HSCs, and we speculate on the ability of Human T-cell lymphotropic virus type 1 (HTLV-1) to generate an "infectious" leukemic/cancer stem cell (ILSC/ICSC).

## What Defines a HSC?

HSCs are pluripotent stem cells that can generate all hemato-lymphoid cells. A cell must meet four basic functional requirements to be defined as a HSC: 1) the capability for self-renewal, 2) the capability to undergo apoptosis, 3) the maintenance of multilineage hematopoiesis, and 4) the mobilization out of the bone marrow into the circulating blood. The ability of HSCs to permanently reconstitute an irradiated recipient host is the most stringent test to evaluate if a population is a true HSC. Long-term transplantation experiments suggest a clonal diversity model of HSCs where the HSC compartment consists of a fixed number of different types of HSCs, each with an epigenetically preprogrammed fate. The HP/HSC population is typically defined by surface expression of CD34 and represents a heterogeneous cell population encompassing stem cells, early pluripotent progenitor cells, multipotent progenitor cells, and uncommitted differentiating cells [8]. HSCs have the potential to proliferate indefinitely and can differentiate into mature hematopoietic lineage specific cells.

**Figure 1:**
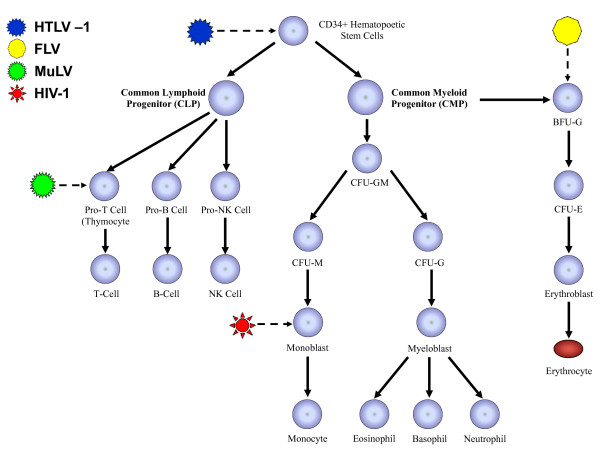
**Hematopoiesis and retroviral infection**: CD34^+ ^hematopoietic stem cells (HSCs) can undergo self-renewal as well as undergoing maturation to give rise to common lymphoid progenitor (CLP) and common myeloid progenitor (CMP) cells, which serve as precursors to all lymphoid and myeloid cells respectively. HSCs as well as other lineage specific progenitors are permissive for infection by a variety of murine and human retroviruses including HIV-1 and HTLV-1.

In adults, HSCs are maintained within the bone marrow and differentiate to produce the requisite number of highly specialized cells of the hematopoietic system. HSCs differentiate into two distinctive types of hematopoietic progenitors: 1) a common lymphoid progenitor (CLP) population that generates B-cells, T-cells and NK cells, and 2) a common myeloid progenitor (CMP) population that generates granulocytes, neutrophils, eosinophils, macrophages and erythrocytes (Figure 1). Lineage commitment of these progenitors involves a complex process that can be induced in response to a variety of factors, including the modulation of hematopoietic-associated cytokines and transcription factors. These factors serve dual purposes both by maintaining pluripotency and by actively inducing lineage commitment and differentiation of HSCs [9-18]

## Leukemia Stem Cells/Cancer Stem Cells (LSC/CSC)

The cancer stem cell hypothesis postulates that cancer can be initiated, sustained and maintained by a small number of malignant cells that have HSC-like properties including self-renewal and pluripotency [19-21]. The hierarchical organization of leukemia was first proposed by Fialkow et al. in the 1970s, and it was later demonstrated that acute myeloid leukemia (AML) contains a diversity of cells of various lineages but of monoclonal origin [22]. It is now well established that HSCs are not only responsible for the generation of the normal hematopoietic system but can also initiate and sustain the development of leukemia, including AML [2,7,23]. This hematopoietic progenitor, termed a leukemic/cancer stem cell (LSC/CSC), is the result of an accumulation of mutations in normal HSCs that affect proliferation, apoptosis, self-renewal and differentiation [24]. One of the most well established models for this theory came from the seminal work of John Dick and colleagues that established cancer stem cells at the top of a hierarchical pyramid for the establishment of AML [25]. Many signaling pathways, such as the *Wnt *signaling pathway, that have been classically associated with solid cancers are now also associated with HSC development and disease [26,27]. CSCs have been unequivocally identified in AML and are also suspected to play a role in other leukemias, including chronic myelogenous leukemia (CML) and acute lymphoblastic leukemia (ALL) [28-30].

In order to be defined as a LSC/CSC, cells must have the ability to generate the variety of differentiated leukemic cells present in the original tumor and must demonstrate self-renewal. The classical experiment to define a cancer stem cell is its ability to reproduce the disease phenotype of the original malignancy in immunocompromised mice. LSC/CSC have the ability to recapitulate the original disease phenotype following transplantation into NOD/SCID mice as illustrated by the transplantation of CD34^+^CD38^- ^LSC/CSC obtained from AML patients [25,31,32]. Interestingly, the CD34^+^CD38^- ^cell surface phenotype of LSC/CSC is shared by immature hematopoietic precursors including HSCs, raising the possibility that LSC/CSC arise from HSCs. Indeed, the transplantation of mature CD34^+^CD38^+ ^cells fails to recapitulate AML in NOD/SCID mice indicating that the HSC rather than the more mature CD34^+^CD38^+ ^progenitor cell, is the LSC/CSC. The identification and characterization of LSC/CSC is critical for designing specific therapies since LSC/CSCs are relatively resistant to traditional radiation and chemotherapy [33-35]. This theory provides an attractive model for leukemogenesis because the self-renewal of HSCs allows for multiple genetic mutations to occur within their long life span. For HSCs to become LSC/CSC, fewer genetic mutations may be required than in mature hematopoietic cells, which must also acquire self-renewal capacity [36].

## The Cancer Stem Cell Hypothesis

**Figure 2:**
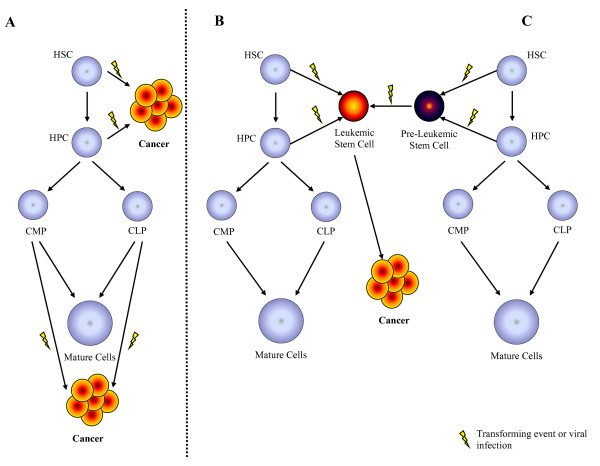
**Generation of Leukemic Stem Cells**. Three hypotheses have been proposed that lead to the development of leukemic stem cells (LSC/CSC): (A) LSC/CSC might arise from either a hematopoietic stem cell (HSC), hematopoietic progenitor cell (HPC), committed lymphoid progenitor (CLP) or committed myeloid progenitor (CMP), (B) from a multipotent HSC or HPC into LSC/CSC through a single transformation event or, **(C) **from HSC or HPCs through a series of transformation events initiated by the generation of a pre-LSC/CSC.

There are currently three hypotheses that address the question of which target cell in cancer undergoes leukemic transformation (Figure 2) [34]. The first hypothesis proposes that multiple cell types within the stem and progenitor cell hierarchy are susceptible to transformation. Mutational events alter normal differentiation patterns and promote clonal expansion of leukemic cells from a specific differentiation state. The second hypothesis proposes that the mutations responsible for transformation and progression to leukemia occur in primitive multipotent stem cells and result in the development of a LSC/CSC. Thus, disease heterogeneity results from the ability of the LSC/CSC to differentiate and acquire specific phenotypic lineage markers [37]. The final hypothesis proposes that progression to acute leukemia may require a series of genetic events beginning with clonal expansion of a transformed LSC/CSC. This "two-hit" model of leukemogenesis suggests that there is a pre-leukemic stem cell that has undergone an initial transformation event, but has not yet acquired the additional mutations necessary to progress to leukemia [38].

Deregulation of genes involved in normal HSC self-renewal and differentiation in human cancer suggests an overlap in the regulatory pathways used by normal and malignant stem cells. Emerging evidence suggests that both normal and cancer stem cells share common developmental pathways. Since the signaling pathways that normally regulate HSC self-renewal and differentiation are also associated with tumorigenesis, it has been proposed that HSCs can be the target for transformation in certain types of cancer [20]. HSCs already have the inherent ability for self-renewal and persist for long periods of time in comparison to the high turnover rate of mature, differentiated cells. HSCs possess two distinctive properties that can be deregulated to initiate and sustain neoplastic malignancies, namely self-renewal and proliferation. Retroviral infection in HSCs may therefore result in the accumulation of mutations and in the modulation of key hematopoiesis-associated gene expression patterns. The alteration of normal hematopoietic signaling pathways, including those related to self-renewal and differentiation, may lead to the generation of a LSC/CSC population. During normal hematopoiesis, the HSC undergoes self-renewal or enters a committed, lineage specific differentiation and maturation pathway. Once HSCs commit to a lineage specific pathway and become terminally differentiated, they lose the capacity to undergo self-renewal [39,40]. LSC/CSC however can undergo long-term proliferation without entering terminal differentiation resulting in the manifestation of hematological malignancies.

## Retroviral Infection and Hematopoiesis

Recent evidence suggests that viral infection may have a profound influence on normal hematopoiesis [41]. Viral infection of HP/HSCs may adversely affect the levels of cytokines and transcription factors vital for proliferation and differentiation. Alternatively, viral infection may induce cytolysis, apoptosis and/or the destruction of progenitor cells, resulting in perturbation of hematopoiesis. Additionally, infected HPCs may differentiate resulting in dissemination of pathogens into diverse anatomical sites and to an effective spread of infection.

HP/HSCs can also serve as targets for cellular transformation by specific viruses partly because of their innate ability for self-renewal. CD34^+ ^HP/HSCs are susceptible to infection with a number of viruses including HIV-1, HTLV-1, Hepatitis C virus, JC virus, Parvovirus, Human Cytomegalovirus (HCMV), and the Human Herpesviruses (HHV): HHV-5, HHV-6, HHV-7, HHV-8 [3-5,42-52]. The concept that viruses can invade, infect and establish a latent infection in the bone marrow was first demonstrated in studies with HCMV. HCMV infects a variety of cell types, including hematopoietic and stromal cells of the bone marrow, endothelial cells, epithelial cells, fibroblasts, neuronal cells, and smooth muscle cells [3,53-57]. The bone marrow is a site of HCMV latency [5,58], but the primary cellular reservoir harboring latent virus within the bone marrow is controversial. Latent viral genomes are detected in CD14^+ ^monocytes and CD33^+ ^myeloid precursor cells [59,60]. However HCMV can also infect CD34^+ ^hematopoietic progenitor populations, and viral DNA sequences can be detected in CD34^+ ^cells from healthy seropositive individuals [45,46,58,61], suggesting that a primitive cell population serves as a renewable primary cellular reservoir for latent HCMV. The finding that HCMV DNA sequences are present in CD34^+ ^cells of seropositive individuals is consistent with the hypothesis that HCMV resides in a HPC which subsequently gives rise to multiple blood cell lineages. Recently, it has also been proposed that other viruses such as HTLV-1 and Kaposi's Sarcoma Herpesvirus (KSHV) can also infect CD34^+ ^HP/HSCs and establish latent infection within the BM resident cells [52,62].

Apart from the establishment of latent infection within the bone marrow (BM), suppression of hematopoiesis has been documented to occur following infection of HPCs with HCMV, HHV-5, HHV-6, HIV-1, and measles virus either as a result of direct infection of HPCs or by indirect mechanisms such as disruption of the cytokine milieu within the stem cell niche following infection of bone marrow stromal cells. Our laboratory has reported that HTLV-1 and KSHV infection of CD34^+ ^HP/HSCs suppresses hematopoiesis *in vitro *and that viral infection can be disseminated into mature lymphoid cell lineages *in vivo *when monitored in humanized SCID mice (HU-SCID) [52,63,64]. HTLV-1 and KSHV are both associated with hematological malignancies and it is plausible that CSCs can be generated following infection of HP/HSCs with these viruses.

Multiple retroviruses establish latent infections in HP/HSCs resulting in perturbation of hematopoiesis and induction of viral pathogenesis [65-69]. Retroviral infections of HSCs can have adverse effects including induction of cell-cycle arrest and increased susceptibility to apoptosis, both would manifest in the suppression of hematopoiesis. Additionally, mutations and transcriptional deregulation of specific hematopoiesis-associated genes can skew normal hematopoiesis toward specific lineages and have been demonstrated to occur following infection of HP/HSCs with HIV-1, HTLV-1 and Friend Leukemia virus (FLV) [64,70,71].

Hematopoiesis occurs in the bone marrow microenvironment, a complex system comprised of many cell types including stromal cells that produce cytokines, growth factors and adhesion molecules vital for the maintenance, differentiation and maturation of HP/HSCs [9,11]. Apart from infection of HSCs, retroviruses such as HIV-1 and Moloney Murine leukemia virus (M-MuLV) have been shown to infect bone marrow stromal cells, compromising their ability to support hematopoiesis and resulting in multilineage hematopoietic failure [72,73].

## Retroviruses and Leukemogenesis: The "two-hit" Hypothesis

**Figure 3:**
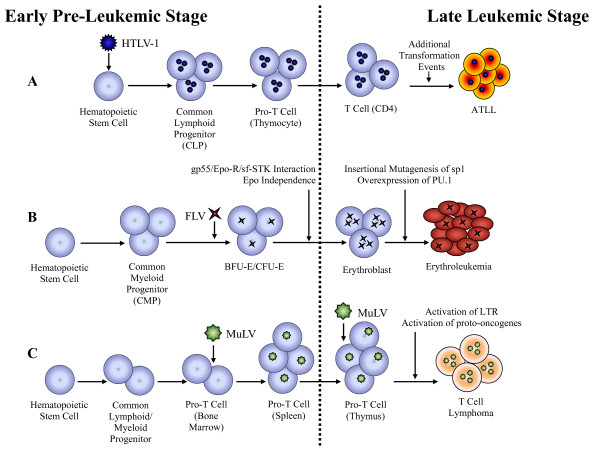
**The "Two-Hit" Model of Retrovirus-Induced Leukemogenesis**. (A) HTLV-1 infection of CD34^+ ^hematopoietic progenitor and stem cells (HP/HSCs) leads to the development of Adult T-cell leukemia/lymphoma (ATLL). (B) FLV infection of erythroid progenitors leads to erythroleukemia. (C) M-MuLV infection of pro-T cells leads to T-cell lymphoma. The dotted line indicates the separation between the early and late phase of infection.

Studies of retroviral induced leukemia have proven very useful in understanding the multi-step processes associated with leukemogenesis. Moreover, these models have broadened our understanding of hematopoiesis and hematopoietic stem cell biology. Retroviral infection models such as FLV and M-MuLV, which induce leukemic states in mice, have emerged as powerful tools to study the molecular mechanisms associated with leukemogenesis and the generation of LSC/CSCs [74-78]. The emerging concept from these murine models is that acute leukemia arises from cooperation between two distinctive mutagenic events; one interfering with differentiation and another conferring a proliferative advantage to HP/HSCs (Figure 2C) [79,80]. Studies from Avian Erythroblastosis virus (AEV), FLV and M-MuLV-induced leukemia/lymphoma models demonstrate that leukemia/lymphoma development depends on: (1) a mutation that impairs differentiation and blocks maturation, (2) a mutation that promotes autonomous cell growth, and (3) that neither mutational event is able to induce acute leukemia by itself [68,81]. Thus, these models provide direct evidence for the "two-hit model" of leukemogenesis as has been proposed for some LSC/CSC induced hematological malignancies, including AML [79]. This concept is perhaps best illustrated by AEV infection in birds, FLV and MuLV infection in mice and in HTLV-1 infection in humans (Figure 3).

During AEV infection, the oncogenic tyrosine kinase v-*Erb-b*, together with the aberrant nuclear transcription factor *v-Erb-A *are transduced. The mutated thyroid hormone receptor α, *v-Erb-A*, becomes unresponsive to the ligand and actively recruits tyrosine kinases. These kinases, such as stem-cell factor activated c-kit, cause arrest of erythroid differentiation at the BFU-E/CFU-E stage. Additionally, *v-Erb-b *encodes a mutated epidermal growth factor receptor that induces extensive erythroblast self-renewal [69,82]. These two virally-induced events promote the abnormal proliferation of erythroid progenitors and lead to the development of leukemia.

Another relevant leukemogenesis model induced by retroviral infection of HPCs is acute erythroleukemia caused by the infection of mice with FLV [83-85]. FLV has two distinct viral components, a replication-competent Friend Murine Leukemia virus (F-MuLV) and a replication defective pathogenic component known as the Friend Spleen Focus Forming virus (F-SFFV) [85-87]. The pathogenic component of FLV (F-SFFV) can infect a variety of hematopoietic cells, though early erythroid progenitors are the primary target for infection [86,88]. F-SFFV can alter the normal growth and differentiation profile of erythroid progenitor cells leading to leukemogenesis. The induction of multistage erythroleukemia by FLV is also a two stage process: a pre-leukemic stage known as "erythroid hyperplasia" and a leukemic phase referred to as "erythroid cell transformation" (Figure 3B). The pre-leukemic stage is characterized by the infection and random integration of F-SFFV virus into erythroid precursor cells, forming an infected stem cell population, followed by the expression of the viral envelope glycoprotein gp55 on the cell surface. gp55 subsequently binds to the cellular receptor of erythropoietin (Epo-R) and interacts with the sf-Stk tyrosine kinase signaling pathway leading to a constitutive activation signal for the proliferation of undifferentiated erythroid progenitor cells independent of erythropoietin [83,89,90]. Within the proliferating erythroid progenitor cell population are infected cells with randomly integrated virus in the *sp-1 *locus, which leads to the activation and overexpression of *PU.1*. Originally isolated by Moreau-Gachelin and co-workers as a gene targeted for recurrent insertions of SFFV, *PU.1 *has subsequently been shown to be involved in terminal myeloid differentiation, B and T-cell development, as well as maintenance of normal erythropoiesis and HSC development [91,92]. The over-expression of *PU.1 *in erythroid precursor cells as a result of SFFV integration leads to a block in erythroid differentiation and, in conjunction with the inactivation of p53, clonal expansion of these leukemic cells in susceptible mice [71,91]. Thus FLV-mediated erythroleukemia is associated with two distinctive phases, "the pre-leukemic phase" mediated by gp55 binding to Epo-R and the "leukemic phase" mediated by SFFV integration and the subsequent over-expression of *PU.1*. This demonstrates that both AEV and FLV infection follow the two-hit model of the cancer stem cell hypothesis.

M-MuLV is a non-acute retrovirus that typically induces a T-cell lymphoma after a latency period of 3-6 months [67]. The tumor cells typically have the phenotype of immature T-cells (CD4^-^/CD8^- ^or CD4^+^/CD8^+^) although some tumors show a more mature surface phenotype (CD4^+^/CD8^-^or CD4^-^/CD8^+^) [72,93]. This led to the hypothesis that the virus might originally infect an immature T-cell or a HPC to form a ICSC/ILSC which then continues to differentiate post-infection, initially in the bone marrow and then in the thymus [67,94]. Because T-lymphocytes develop in the thymus from bone marrow-derived immature precursors (pro-thymocytes), it has been proposed by several investigators that a bone marrow-thymus axis plays an important role in the development of T-cell lymphoma by M-MuLV [93,95-97]. Although the identity of the initial target cell for M-MuLV infection is still unknown, a two-stage leukemogenesis model for the development of M-MuLV-induced leukemia has been proposed [67]. In this model the animal is infected with MuLV on two separate occasions; the first infection occurs in the bone marrow at the pre-leukemic (early) phase which leads to hyperplasia and migration of infected lymphoid progenitors into the thymus where a subsequent infection leads to insertional activation of proto-oncogenes and outgrowth of the tumor resulting in the leukemic (late) phase of infection (Figure 3C). Early infection of the bone marrow is thought to be essential for establishment of the pre-leukemic state and for development of spleen hyperplasia. The late phase splenic hyperplasia is the result of a compensatory hematopoiesis due to diminished normal hematopoiesis in the bone marrow resulting from the establishment of the preleukemic phase and plays an integral role in the establishment of malignancy [98-100].

Bovine leukemia virus (BLV) is a deltaretrovirus which causes leukemia/lymphoma in cattle [101] (reviewed by [102,103]) and has been used as a model of HTLV-1 infection and disease. While B-cells are the primary target of BLV infection in contrast to the T-cell tropism displayed by HTLV-1, BLV-infected B lymphocytes are similarly arrested in G_0_/G_1 _and protected from apoptosis similar to properties demonstrated following HTLV-1 infection HP/HSCs [64,104]. It has been suggested that CD5^+ ^B-cell progenitors are more susceptible to BLV infection [105] and that there is a relationship between the B-cell phenotype and BLV tropism [106]. More recently, the existence of a pre-malignant clone has been proposed. This infected progenitor is detectable early after viral infection and could contribute to both genetic instability and clonal expansion, both characteristics of cancer cells [107]. It can therefore be speculated that the infection of progenitor populations by BLV may result in the establishment of an ILSC/ICSC and subsequent development of leukemia.

Much of the current knowledge about leukemic mechanisms originates with studies on AML. AML is characterized by the uncontrolled self-renewal of hematopoietic progenitors that fail to differentiate normally. Induction of AML is associated with a variety of mutations that can be broadly classified into two distinctive categories; mutations in genes encoding transcription factors involved with hematopoietic regulation and mutations in genes encoding proteins linked to survival and proliferation signaling pathways [74-78,108,109]. Studies in mice have shown that neither type of mutation alone is sufficient for the induction of AML and that cooperative mutagenic events are required for disease initiation [69,79]. The leukemogenesis models of AEV, FLV and MuLV validate this concept and underline the importance of these models for the study of down-stream molecular events associated with these mutagenic events. The emergence of LSC/CSC as a result of these oncogenic events would explain the complexity associated with hematological malignancy development such as AML and CML in humans.

## Human T-cell Leukemia Virus Type-1 (HTLV-1) and Adult T-cell Leukemia/Lymphoma (ATLL)

Human T-cell leukemia/lymphoma virus type-1 (HTLV-1) is the causative agent of Adult T-cell Leukemia/Lymphoma (ATLL), an aggressive CD4^+ ^leukemia/lymphoma [110]. ATLL is a rare T-cell malignancy characterized by hypercalcemia, hepatomegaly, splenomegaly, lymphadenopathy, the presence of a monoclonal expansion of malignant CD4^+^CD25^+ ^T-cells that evolve from a polyclonal population of HTLV-1 infected CD4^+ ^T-cells, and infiltration of lymphocytes into the skin and liver. HTLV-1 causes ATLL in a small percentage of infected individuals after a prolonged latency period of up to 20-40 years [111]. Although HTLV-1 can replicate by reverse transcription during the initial phase of infection, the integrated provirus is effectively replicated during proliferation of infected cells [112]. Typically, HTLV-1 infected cells can persist for decades in patients, and the infected cell population transits from a polyclonal phase into a monoclonal expansion during development and progression to ATLL.

There are four ATLL subtypes; acute, lymphomatous, chronic, and smoldering. The first two subtypes are associated with a rapidly progressing clinical course with a mean survival time of 5-6 months. Smoldering and chronic ATLL have a more indolent course and may represent transitional states towards acute ATLL. Clinical features of ATLL include leukemic cells with multi-lobulated nuclei called 'flower cells' which infiltrate into various tissues including the skin and the liver, abnormally high blood calcium levels, and concurrent opportunistic infections in patients [113,114].

Although considerable progress has been made in understanding ATLL biology, the exact sequence of events occurring during the initial stages of malignancy, including the types of cells infected with HTLV-1, remain unclear. The primary target cells for HTLV-1 infection may not only influence HTLV-1 pathogenesis, but the sequestration of these cells in anatomical sites such as the bone marrow may also allow the virus to effectively evade the primary immune response against infection.

## The Role of HSCs in HTLV-1 Infection and Pathogenesis

It has been previously reported by our laboratory and other investigators that HTLV-1 can infect human HP/HSCs [65,115]. It has been hypothesized that HTLV-1 can specifically induce a latent infection in CD34^+ ^HP/HSCs and can initiate preleukemic events in these progenitor cells [62]. These cells could potentially provide a durable reservoir for latent virus in infected individuals. It has been speculated that HTLV-1 infection of CD34^+ ^HPCs may result in the generation of an ILSC/ICSC and may also induce perturbation of normal hematopoiesis, ultimately resulting in the outgrowth of malignant clones and the development of ATLL.

The development of ATLL correlates with neonatal or perinatal transmission of HTLV-1. HTLV-1 carries no cellular proto-oncogenes, and the oncogenic potential of the virus is linked to Tax1, a 40 kDa protein that functions as a trans-activator of viral gene expression and as a key component of HTLV-1-mediated transformation [116,117]. Tax1 is a relatively promiscuous transactivator of both viral and cellular gene transcription and has been closely linked to the initiation of leukemogenesis. Apart from regulating viral gene expression through the 5' long terminal repeat (LTR), Tax1 can modulate the expression of a large variety of cellular genes and proteins including those encoding cytokines, apoptosis inhibitors, cell cycle regulators, transcription factors, and intracellular signaling molecules [116,118-120]. Tax1 usually induces cellular gene expression by the activation of transcription factors such as NF-κB and cyclic AMP response element-binding protein/activating transcription factor (CREB/ATF) [121]. Tax1 has also been shown to *trans*-repress transcription of certain cellular genes, including *bax *[122], human β-polymerase [119], cyclin A [123], *lck *[124], MyoD [125], INK4 [126], and p53 [127].

Transgenic mouse models of Tax1 expression have resulted in the generation of murine malignancies, including a mature T-cell malignancy, underlying the critical role of Tax1 in the manifestation of T-cell leukemia [128,129]. Transgenic mice constructed to target expression of Tax1 to both immature and mature thymocytes using a Lck (Leukocyte-specific protein tyrosine kinase) promoter reproducibly develop immature and mature T-cell leukemia/lymphomas with immunological and pathological similarities to human ATLL [128,129]. In a recent study by Yamazaki et al., splenic lymphomatous cells were harvested and purified from Tax-transgenic mice using a combination of immunological and physiological markers for CSCs and were injected into NOD/SCID mice using a limiting-dilution assay [6]. Injection with as few as 1 × 10^2 ^CSCs was sufficient to recapitulate the original lymphoma and reestablish CSCs in recipient NOD/SCID mice implicating a role for LSC/CSC in the establishment of ATLL.

LSC/CSCs have the ability to self-renew, are sequestered in the bone marrow microenvironment and are relatively resistant to conventional chemotherapeutic treatment regimens. The recent focus and characterization of the role of LSC/CSC in the induction of AML has generated a paradigm for LSC/CSC-generated cancers and has resulted in a re-evaluation of therapeutic strategies for successful targeting and elimination of leukemic cells in patients [31]. Although the Tax-transgenic mouse model is not a complete representation of ATLL manifestation in humans, this finding is intriguing particularly since other investigators have suggested that HTLV-1 infection in the human bone marrow and in human HP/HSCs specifically, may facilitate the early events initiating ATLL development [62]. Since a limited number of ATLL cases display phenotypes indicative of immature hematopoietic cells, HTLV-1 infection and transformation of HP/HSCs in humans may result in the generation of virally-infected ATLL LSC/CSC [130]. Lymphoma cells and LSC/CSC from Tax-transgenic mice were also demonstrated to sequester in the osteoblastic and vascular niches of the bone marrow in transplanted NOD/SCID mice. It is interesting to speculate that if ATLL arises from a LSC/CSC, then the sequestration of HTLV-1-infected HP/HSCs in the bone marrow microenvironment may be a contributing factor in the resistance of this leukemia to treatment with conventional chemotherapies. It remains to be determined if the recent results from the Tax-transgenic model are truly illustrative of the human disease. However, the Tax-transgenic murine model does provide several interesting clues into the mechanisms of HTLV-1 pathogenesis, and this may eventually group ATLL along with other hematological malignancies that have a LSC/CSC origin.

Recapitulating ATLL in 'humanized' SCID (HU-SCID) mice has been challenging, and previous attempts to directly infect mature human T-cells in the human thymus-liver conjoint organ in HU-SCID mice with HTLV-1 failed to induce a malignancy [65]. Recent data from our laboratory demonstrates that *ex vivo *infection of CD34^+ ^HP/HSCs with HTLV-1 reproducibly and consistently results in development of a CD4^+ ^T-cell lymphoma in HU-SCID mice [131]. Clearly, HTLV-1 infection of HP/HSCs plays a pivotal role in the initiation and accelerated progression of malignancy during the course of HTLV-1 pathogenesis.

## HTLV-1 Infected CD34^+ ^HP/HSCs: Notch, PU.1 and micro-RNA Deregulation

Manifestation of ATLL in patients generally occurs decades after infection, suggesting that HTLV-1 latently infects bone marrow stem cells that are sequestered from immunological surveillance. It is conceivable that the initiation of leukemogenesis in HSCs involves the generation of a CSC/LSC that will eventually manifest into the monoclonal ATLL malignancy. Several pathways that regulate HSC self-renewal are also associated with human cancers, including hematopoietic malignancies such as T-cell leukemia [132] and T-ALL [133,134]. It has previously been shown that disruption of normal HSC self-renewal signaling pathways can induce hematopoietic neoplasms [132,135]. Two main reasons suggest that HSCs can serve as target cells for virally-induced leukemia/lymphoma. First, stem cells have constitutively activated self-renewal pathways, requiring maintenance of activation in contrast to the *de novo *activation required in a more differentiated cell. Second, self-renewal provides a persistent target for repeated viral infection and/or continual replication of integrated proviral DNA. HTLV-1 infection of CD34^+ ^HP/HSCs deregulates normal HSC self-renewal pathways through a variety of potential mechanisms suggesting that HTLV-1 infection may generate ILSC/ICSC.

The Notch signaling pathway regulates self-renewal and differentiation of HSCs and has been implicated as a key regulator of human T and B-cell derived lymphomas [135,136]. Studies using adult bone marrow transplantation into NOD/SCID mice demonstrate that inactivation of Notch1 arrests T-cell development at the earliest precursor stage [134] and promotes B-cell development in the thymus [137]. The modulation of Notch levels in LSC/CSC derived from Tax-transgenic mice suggests that Notch may contribute in the development of ATLL similar to its role in other T-cell malignancies such as T-ALL [133,134].

The *sp1 *gene encodes for the transcription factor *PU.1*, which is a member of the ets family of transcription factors, is expressed at various levels in all hematopoietic cells. *PU.1 *expression has been shown to play an important role in the regulation of hematopoiesis [138,139]. Specifically, expression of *PU.1 *is tightly controlled in HSCs and regulates the fate of cells differentiating into lymphocyte, macrophage or granulocyte lineages [140,141]. Deregulation of *PU.1 *expression has been linked to the development of hematopoietic malignancies including the transformation of myeloid cells [92]. During hematopoiesis, *PU.1 *is required for hematopoietic development along both the lymphoid and myeloid lineages, but is down-regulated during erythropoiesis. In AML patients, mutations in Flt3 decrease *PU.1 *expression and block differentiation [141] while mutations in *PU.1 *impair development within both myeloid and lymphoid lineages [142]. Knockout mouse studies have shown that perturbation of *PU.1 *expression results not only in the loss of B-cells and macrophage development, but also delays T lymphopoiesis [143,144]. Additionally, *PU.1 *supports the self-renewal of HSCs by regulating the multilineage commitment of multipotent progenitors, thereby maintaining a pool of pluripotent HSCs within the bone marrow [145,146].

Notably the reduction in *PU.1 *expression in bone marrow derived CD34^+ ^HP/HSCs has been shown to induce an intermediate stage of poorly differentiated pre-leukemic cells which, with the accumulation of additional genetic mutations, results in an aggressive form of AML [147]. The HTLV-I accessory protein p30 has also been shown to interact with the *ets *domain of *PU.1 *resulting in impairment of the DNA binding activity of *PU.1 *and subsequent inhibition of *PU.1*-dependent transcription [148]. HTLV-1 p30-mediated alteration of *PU.1 *expression may be a contributing factor in the deregulation of hematopoiesis due to HTLV-1 infection of HSCs and may contribute to the establishment of ILSC/ICSC.

Bmi-1 (B-lymphoma Mo-MuLV insertion region), which belongs to the polycomb group of epigenetic chromatin modifiers, was originally identified as an oncogene [149]. Bmi-1 is required for the maintenance of HSC self-renewal in mice and is also involved in regulation of genes controlling cell proliferation, survival and differentiation of HSCs [149-152]. Deficiency of Bmi-1 results in a progressive loss of HSCs and in defects in the stem cell compartment of the nervous system [153]. Bmi-1 expression is elevated in HP/HSCs in contrast to differentiated hematopoietic cells, and both self-renewal as well as the *in vivo *repopulation potential of HSCs is dependent on Bmi-1 [152,154-156]. It has been reported that Bmi-1 is required for the activation and survival of pre-T-cells and during transition from DN to DP T-cells [157]. Bmi-1 is required for the proliferation of LSC/CSCs, and the deregulation of Bmi-1 is linked to human cancers [155,158]. Notably LSC/CSCs from Tax1-transgenic mice show a robust down-regulation of Bmi-1, providing a mechanistic link between HTLV-1 infection and deregulation of hematopoiesis.

Micro-RNAs (miRNAs) are a class of non-coding RNAs, 20-25 nucleotides long, that play an important role in both normal and malignant hematopoiesis, including self-renewal, differentiation and lineage specificity of HPCs [159-163] (reviewed in [164]). Loss of miRNAs has also been reported in a variety of cancers indicating that alteration of miRNA levels might play a critical role in tumorigenesis [165-167]. miR-150 is preferentially expressed in the megakaryocytic lineage and has been recently shown to drive the differentiation of megakaryocyte-erythrocyte precursors toward megakaryocyte development at the expense of erythroid differentiation [168]. Over-expression of miR-221 and miR-222 interferes with the *kit *receptor and blocks engraftment of HSCs in humanized mice [169]. Over-expression of miRNA-181a has been linked to the development of AML and CLL [170,171]. These studies highlight the role of miRNA in regulating normal hematopoiesis and suggest that miRNA expression may modulate the manifestation of hematopoietic malignancies.

Retroviruses such as HIV-1 and HTLV-1 have been recently shown to target miRNAs for modulation of key cellular pathways including cell-cycle regulation and immune responses [172-174]. Specifically, miRNAs that are involved in the regulation of cell proliferation, apoptosis and immune responses are up-regulated in ATL cells [175,176]. Bellon et al. recently demonstrated that miRNAs involved in normal hematopoiesis and immune responses are also profoundly deregulated in ATLL cells indicating a possible link between modulation of cellular miRNA expression and deregulation of hematopoiesis by HTLV-1 [177]. Specifically, significant changes in the expression of miR-223 and miR-150 in ATLL patient samples were identified. miR-223 controls the terminal differentiation pathway of HSCs and is upregulated following differentiation into myeloid and lymphoid progenitors [162]. The differential expression of miR-150 regulates lineage decision between T and B-cells. Ectopic expression of miR-150 in lymphoid progenitors enhances T lymphopoiesis with respect to B lymphopoiesis [178]. The deregulation of cellular miRNAs might contribute to the transformation process resulting in the development of ATLL.

HTLV-1 infection in HP/HSC could result in aberrant miRNA expression ultimately predisposing HSC development toward T lymphopoiesis. Since expression of these miRNAs (223 and 150) are restricted to HP/HSCs, CLPs and CMPs, patient derived primary ATLL cells may originated from an infected HPC population in contrast to *in vitro*-established HTLV-1 infected CD4^+ ^T cell lines. This supports the hypothesis that ATLL cells are derived from HTLV-1 infected CD34^+ ^HP/HSCs rather than virally transformed mature T-cells [64,128,129]. Upon differentiation of an HTLV-1 infected CD34^+ ^HPC, the alteration of miRNA levels may favor T-cell differentiation, as recently demonstrated by the exclusive development of CD4^+ ^mature T-cell lymphomas in HU-SCID mice reconstituted with CD34^+ ^HPCs infected *ex vivo *with HTLV-1[131].

## Tax1 and Cell Cycle Regulation in HP/HSCs

HTLV-1 Tax1 has been shown to induce G_0_/G_1 _cell cycle arrest leading to quiescence in both cultured mammalian cell lines and primary human CD34^+ ^HPCs [116,179,180]. Likewise, the expression of Tax1 in *Saccharomyces cerevisiae *leads to growth arrest and loss of cell viability [181,182]. Intriguingly, in addition to increasing the levels of cyclins and CDKs, Tax1 also increases the levels of CDK inhibitors p16^Ink4^, p21^cip1/waf1 ^(p21) and p27^kip ^(p27) in infected cells [63,64,179,183,184]. Over-expression of p21 inhibits two critical checkpoints in the mammalian cell cycle, namely G_1_/S and S/G_2_, through p53-independent and dependent pathways [185]. Moreover, p21 and p27 are the key contributors in the cell-cycle regulation of CD34^+ ^HPCs [186-188]. Tax1 has also been shown to suppress human mitotic checkpoint protein MAD1 resulting in deregulation of the G2/M phase of the cell cycle resulting in aneuploidy [189]

Cell cycle progression is highly regulated in CD34^+ ^HPCs with a majority of CD34^+ ^HPCs residing in quiescence and demonstrating a unique expression pattern of CDKs, cyclins, and CDK inhibitors. The CDK inhibitors p21 and p27, in particular, have been shown to be key contributors in restricting cell cycle entry from G_0 _and maintaining quiescence in CD34^+ ^HPCs [186-188]. We have previously shown that during HTLV-1 infection, induction of G_0_/G_1 _cell cycle arrest and suppression of multilineage hematopoiesis in HPCs is attributed to the concomitant activation of p21 and p27 in these cells by Tax1 [63,64,180]. Although Tax1 usually induces cellular gene expression by activation of transcription factors such as NF-κB, CREB/ATF and Akt [190], it has recently been suggested that Tax1 deregulation of p21 and p27 may also be mediated independently of NF-κB activation [191] and p53 [184]. Moreover, the reported absence of NF-κB activity in CD34^+^CD38^- ^HSCs [192] suggests that HP/HSCs provide a unique microenvironment for HTLV-1 infection which stands in stark contrast to the cellular environment provided by mature CD4^+ ^T lymphocytes. It may be inferred that Tax1-mediated cell cycle deregulation is cell-type specific, inducing cell cycle arrest in HPCs while concurrently maintaining the ability to activate cell proliferation in mature CD4^+ ^T-lymphocytes.

**Figure 4:**
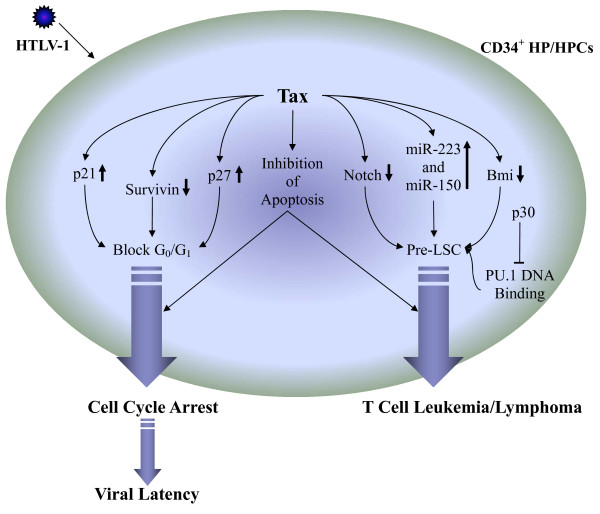
**The Role of HTLV-1 Infection of HSCs: Potential Mechanisms for Generation of an Infectious Leukemic Stem Cell (ILSC/ICSC)**. HTLV-1 infection and subsequent Tax1 expression can lead to either cell cycle arrest or generation of pre-leukemic stem cells (pre-LSC/CSC) from infected CD34^+ ^hematopoietic progenitor and stem cells (HP/HSCs).

Survivin, originally identified as a member of the inhibitor of apoptosis protein family, has recently been implicated in regulating hematopoiesis, cell cycle control and transformation [193-196]. Survivin is expressed in normal adult bone marrow cells and in CD34^+ ^HPCs where it regulates proliferation and/or survival, and survivin expression is upregulated by hematopoietic growth factors [197]. Notably, survivin has been shown to be a key mediator of early cell cycle entry in CD34^+ ^HPCs and regulates progenitor cell proliferation through p21-dependent and independent pathways [198], in addition to regulating apoptosis of HSCs [199]. This implicates survivin as an integral cellular factor, regulating multiple aspects of hematopoiesis. HTLV-1 mediated suppression of hematopoiesis in CD34^+ ^HPCs is regulated, in part, by down-regulation of survivin expression in these cells by Tax1 [64]. Notably, CD34^+^CD38^- ^HSCs demonstrate elevated sensitivity to cell-cycle arrest following HTLV-1 infection in comparison to more mature CD34^+^CD38^+ ^HPCs, suggesting that HTLV-1 may target stem cells to facilitate a latent infection *in vivo *by inducing cell cycle arrest to induce cellular quiescence (Figure 4).

## HTLV-1 Interaction with CD34^+ ^HP/HSCs: Emerging Views

Emerging evidence has led to a new view of HTLV-1 mediated leukemogenesis that correlates neonatal transmission of HTLV-1 with viral infection targeting of HP/HSCs and immature human thymocytes [63,65,115]. This hypothesis challenges the current view that mature differentiated CD4^+ ^T-cells are the exclusive target for HTLV-1 infection and for the initiation of ATLL. Analysis of bone marrow samples from pediatric HTLV-1 infections would confirm the hypothesis that HTLV-1 infection enters and is sequestered in the CD34^+^HP/HSCs in the bone marrow. It is noteworthy that previous reports have demonstrated HTLV-1 transmission following a bone marrow transplantation procedure from a HTLV-1 infected donor [200]. HTLV-1 infection of HP/HSCs can result in skewing of hematopoiesis toward distinct cellular lineages and outgrowth of malignant clones leading to ATLL. HP/HSCs may be critical target cell for HTLV-1 infection and for establishment of latency *in vivo *providing a reservoir of infected cells which progresses, after the accumulation of additional molecular events, to the development of ATLL [62,65]. This hypothesis is supported by recent identification of a rare CSC population in Tax-transgenic mice [6,128]. Notably, our laboratory has detected a high incidence of HTLV-1 proviral sequences in CD34^+ ^HP/HSCs from HTLV-1-infected patient peripheral blood lymphocyte samples, suggesting that HP/HSCs are a natural cellular reservoir for HTLV-1 infection [131]. The down-regulation of key hematopoietic genes, including Notch1 and Bmi-1, in CSCs from Tax-transgenic mice indicates that the CSC potentially emerges from primitive HPCs or immature thymocytes and highlights the role of Tax1 expression in the induction of lymphoproliferative disease (Figure 4). The role of HTLV-1 Tax in HP/HSCs includes cell cycle deregulation and perturbation of hematopoiesis, as we have previously reported [63,180]. Clearly many parameters defining how HTLV-1 and its associated viral genes (including Tax1, p30 and HBZ [201]), may contribute to the development of a ILSC/ICSC in ATLL have yet to be established. The role of the HTLV-1 antisense encoded protein HBZ is of particular interest as it is consistently expressed in all ATLL patient cells examined in contrast to Tax1 which is usually silenced in ATLL cells [202,203]. Emerging *in vivo *murine models, particularly the HU-SCID mouse models, will help characterize the pathobiology of HTLV-1 infection and establish the existence of ILSC/ICSC. Moreover, these models will allow for the identification of events resulting in leukemia-initiation and progression and for the pre-clinical therapeutic evaluation for this fatal malignancy which currently lacks effective treatment regimens.

## Perspectives

Retroviral infection of HP/HSCs in the bone marrow clearly provides a reservoir for infected cells and results in dramatically altered patterns of hematopoiesis. Determining and identifying whether retroviruses, such as HTLV-1, exploit this cellular trait to establish an ILSC would present a new paradigm in the pathobiology of HTLV-1 infection and would allow novel targeted treatments to be designed in order to intervene and treat retroviral mediated neoplasms.

## Abbreviations

LSC/CSC: leukemic stem cell/cancer stem cell; HP/HSC: hematopoietic progenitor/stem cell; HTLV-1: human T cell lymphotropic virus type 1; ILSC/ICSC: infectious leukemic/cancer stem cell; CLP: common lymphoid progenitor; CMP: common myeloid progenitor; AML: acute myeloid leukemia; ALL: acute lymphoblastic leukemia; CML: chronic myelogenous leukemia; HU-SCID mouse: humanized severe combined immunodeficient mouse; HIV-1: human immunodeficiency virus type-1; FLV: Friend leukemia virus; M-MuLV: Moloney murine leukemia virus; AEV: Avian erythroblastosis virus; BLV: Bovine leukemia virus; ATLL: Adult T cell leukemia/lymphoma; CREB/ATF: cyclic AMP response element-binding protein/activating transcription factor; Bmi-1: B-lymphoma Mo-MuLV insertion region; DN: double negative; DP: double positive; miRNAs: micro-RNAs.

## Competing interests

The authors declare that they have no competing interests.

## Authors' contributions

PB and LC were responsible for drafting and revising the manuscript as well as organizing the content. ES created Figures 1, 2, 3 and 4 and their legends and proofread the final version of the manuscript for content and consistency. GF assisted in all aspects of writing the manuscript from revisions to final approval of the version to be published. All authors read and approved the final manuscript.
